# Crystal structure of plasmoredoxin, a redox-active protein unique for malaria parasites

**DOI:** 10.1016/j.crstbi.2022.03.004

**Published:** 2022-04-01

**Authors:** Karin Fritz-Wolf, Jochen Bathke, Stefan Rahlfs, Katja Becker

**Affiliations:** a Biochemistry and Molecular Biology, Interdisciplinary Research Center, Justus Liebig University, 35392, Giessen, Germany; b Max-Planck Institute for Medical Research, 69120, Heidelberg, Germany

**Keywords:** *Plasmodium falciparum*, Plasmoredoxin, Thioredoxin, Monomer–dimer population, Disulfide bonds, Antioxidants, Redox, amino acids, aa, glutathione reductase, GR, glutathione (reduced / oxidized), GSH / GSSG, glutaredoxin, Grx, peroxiredoxin Prx, either 2-Cys-Prx or Prx1m, plasmoredoxin, Plrx, thioredoxin reductase, TrxR, Trx, thioredoxin, tryparedoxin reductase, TR, trypanothione, Try (reduced, oxidized), tryparedoxin, Txn, *Human*, *h*, *Plasmodium falciparum*, *Pf*, *Wuchereria bancrofti*, *Wb*, *Leishmania major*, *Lm*, *Trypanosoma brucei*, *Tb*, *Crithidia fasciculata*, Cf, *Bacteroides fragilis*, *Bf*

## Abstract

Plasmoredoxin is a 22 ​kDa thiol–disulfide oxidoreductase involved in cellular redox regulatory processes and antioxidant defense. The 1.6 ​Å structure of the protein, solved via X-ray crystallography, adopts a modified thioredoxin fold. The structure reveals that plasmoredoxin, unique for malarial parasites, forms a new subgroup of thioredoxin-like proteins together with tryparedoxin, unique for kinetoplastids. Unlike most members of this superfamily, Plrx does not have a proline residue within the CxxC redox motif. In addition, the Plrx structure has a distinct C-terminal domain. Similar to human thioredoxin, plasmoredoxin forms monomers and dimers, which are also structurally similar to the human thioredoxin dimer, and, as in humans, plasmoredoxin is inactive as a dimer. Monomer–dimer equilibrium depends on the surrounding redox conditions, which could support the parasite in reacting to oxidative challenges. Based on structural considerations, the residues of the dimer interface are likely to interact with target proteins. In contrast to *human* and *Plasmodium falciparum* thioredoxin, however, there is a cluster of positively charged residues at the dimer interface of plasmoredoxin. These intersubunit (lysine) residues might allow binding of the protein to cellular membranes or to plasminogen. Malaria parasites lack catalase and glutathione peroxidase and therefore depend on their other glutathione and thioredoxin-dependent redox relays. Plasmoredoxin could be part of a so far unknown electron transfer system that only occurs in these parasites. Since the surface charge of plasmoredoxin differs significantly from other members of the thioredoxin superfamily, its three-dimensional structure can provide a model for designing selective redox-modulatory inhibitors.

## Highlights

• Two high resolution X-ray structures – confirmed that Plrx belongs to the thioredoxin superfamily.
• Structure and surface charge differ from the other members of the thioredoxin superfamily.
• The highest relationship in terms from sequence and structural fold is found with tryparedoxins.
• Similar to human thioredoxin, plasmoredoxin forms monomers and dimers.
• Potential as drug target.

## Graphical abstract

**Figure:**
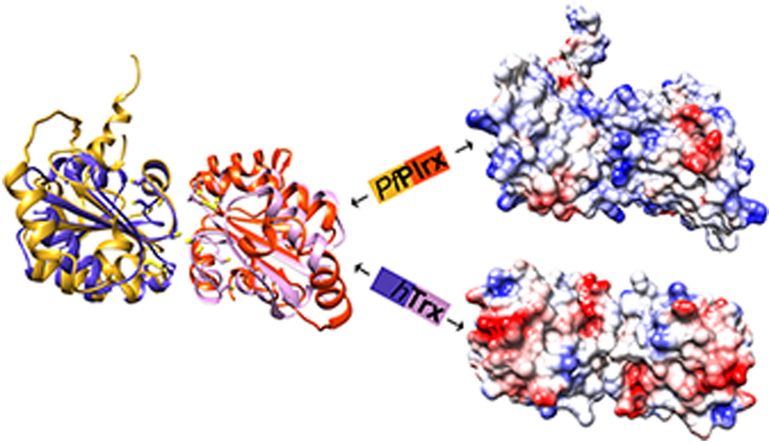

## 1 Introduction

Malaria still causes 400,000 deaths per year; most of them are pregnant women and children less than 5 years old (WHO, 2019). Obviously, new drugs are still urgently needed. Malaria parasites depend crucially on their intercellular redox balance, so proteins involved in antioxidant defense are superb targets for the development of antimalarial drugs. In *Plasmodium*, two redox systems are known for antioxidative defense and redox regulation: a glutathione system comprising NADP, glutathione reductase (GR), glutathione (GSH), glutathione S-transferase, and glutaredoxin (Grx) and a thioredoxin system comprising NADP, thioredoxin reductase (TrxR), thioredoxin (Trx), and thioredoxin-dependent peroxidases (Prx) (Rahlfs et al., 2002; Jortzik and Becker, 2012). In kinetoplastids, such as *Trypanosoma* or *Leishmania*, an alternative system exists to maintain intracellular redox balance. This system comprises trypanothione reductase (TR), trypanothione (Try) and tryparedoxin (Txn) with a terminal electron acceptor such as tryparedoxin-dependent peroxidase or ribonucleotide reductase (Lüdemann et al., 1998; Wagner et al., 2019; Krauth-Siegel et al., 2003).

Becker et al. discovered an unknown redox-active protein that only exists in *Plasmodium* species and was therefore named plasmoredoxin (Plrx) (Becker et al., 2003). Plrx belongs to a novel family of redox-active proteins that are members of the thioredoxin superfamily. This family includes the proteins Trx, Grx, and Txn, which are part of known redox systems in *Plasmodium* and kinetoplastids. Like Trx, Grx, and Txn, Plrx reduces peroxiredoxins (Nickel et al., 2005, 2006) and is active in an insulin reduction assay; furthermore it is probably involved in ribonucleotide reduction and glutathione homeostasis (Becker et al., 2003). A Plrx knockout was not lethal in the rodent malaria parasite *P. berghei* (Buchholz et al., 2008), but the function of Plrx in *P. falciparum* has not yet been investigated.

The oxidoreductases TrxR, GR, and TR reduce thioredoxin, GSSG, and Try_oxidized_, respectively. In the next step, GSH and Try_reduced_ non-enzymatically reduce Grx and Txn (Lüdemann et al., 1998; Alphey et al., 1999). In contrast to the reduction of Trx, two steps are thus needed to reduce Grx and Txn. However, an oxidoreductase or a comparable peroxidase that efficiently reduces *Pf*Plrx has not yet been identified (Becker et al., 2003). Low molecular weight substrates (TCEP, DTT), including trypanothione but not glutathione, are able to reduce *Pf*Plrx. Consequently, a kind of TR/Try system might reduce Plrx, as seen in kinetoplastids; however the respective players are not present in *Plasmodium* and other comparable enzyme/substrate couples fulfilling this function have not been identified so far. Nonetheless, other members of the thioredoxin superfamily such as Txn, Grx, and Trx (in their prereduced form) are able to interact with and transfer electrons to oxidized *Pf*Plrx (Becker et al., 2003).

*Pf*Plrx is significantly larger (22 ​kDa; 179 amino acids) than classic thioredoxins (ca. 100 aa) or glutaredoxins (ca. 110 aa (Yogavel et al., 2014),); only tryparedoxins are similar in size (165 aa (Fiorillo et al., 2012),). The sequence identity of *Pf*Plrx to other members of the thioredoxin superfamily is very low—less than 27% — but the characteristic active site motif (CXXC) of the family, which determines the biological activity of the protein (Arnér and Holmgren, 2000; Holmgren, 2000), is also present in Plrx. All members of the thioredoxin superfamily share a common 3D structure, the thioredoxin fold, characterized by a central four-stranded β-sheet surrounded by three α-helices (Martin, 1995; Creighton, 1997).

In this study, we solved the 3-dimensional structure of recombinant *P. falciparum* plasmoredoxin. In a range of about 100 amino acids, the structure is typical for a member of the thioredoxin family, but the surface charge is different from other members, which implies different binding partners and therefore different tasks. Oligomerization studies showed that *Pf*Plrx exists as both a monomer and dimer, depending on the redox milieu of the environment. Though, crystallographic analysis revealed that *Pf*Plrx would be inactive as a dimer, comparable to the human Trx dimer.

## 2 Results and discussion

### 2.1 Structure determination

We have obtained monoclinic (space group P2_1_) and hexagonal crystals (space group P6_1_) of *Pf*Plrx from *P. falciparum*, both diffracted to 1.6 ​Å. The asymmetric unit of the monoclinic and hexagonal crystal types contained four and one monomers of *Pf*Plrx, respectively. Attempts to solve the structure by the molecular replacement method were not successful. Finally, we combined single anomalous dispersion and molecular replacement methods to solve the monoclinic structure (Adams et al., 2010). Manually rebuilding (Emsley and Cowtan, 2004) and subsequent refinement resulted in a *Pf*Plrx model, comprising residues 12 to 179. Accordingly, we used this model to obtain the hexagonal structure. Data collection and refinement statistics of all datasets are summarized in Table 1.

**Table 1:** Crystallographic statistics.

	Hexagonal	Monoclinic
Space group	P6_1_	P12_1_1
Unit cell parameters		
a, b, c (Å)	74, 74, 65.27	47.75, 122.73, 72.86
α, β, γ (°)	90, 90, 120	90, 104.88, 90
Data collection		
Beamline	ESRF beamline ESD-ID14-4	SLS beam line X10SA
Temperature (K)	100	100
Wavelength (Å)	0.9793	1
Resolution range	19.45–1.63 (1.69–1.63)	46.26–1.60 (1.66–1.60)
Wilson B-factor (Å^2^)	19.9	25.6
Total reflections	249054 (9822)	483467 (45941)
Unique reflections	25145 (2336)	106413 (10618)
Multiplicity	9.9 (4.2)	4.5 (4.3)
Completeness (%)	99.2 (92.2)	99.9 (99.5)
Mean I/σ (I)	33.9 (4)	13.5 (1.7)
R-merge^a^ (%)	5.7 (34.3)	5.2 (87.6)
R-pim^b^ (%)	1.6 (18.0)	2.7 (46.8)
CC1/2 (%)	100 (87.1)	99.8 (68.3)
Molecules per ASU	1	4
Refinement		
R_work_/R_free_^c^ (%)	16.8 (21.4)/20.0 (24.7)	17.8(27.9)/21.0(30.0)
No. of atoms/average B (Å^2^)		
Protein	1391/27.7	5646/33.5
Ligands	30/50.1	224/53.2
Solvent	149/40.5	311/41.9
Non-hydrogen atoms	1570/28.9	6181/34.6
Protein residues	158	648
Ramachandran plot (%)		
Favored (%)	97.44	97.0
Outliers (%)	0	0
RMS deviations		
Bonds (Å)	0.02	0.020
Angles (°)	1.52	1.41
PDB accession code	7AOJ	7AOO

Statistics for the high-resolution shell are shown in parentheses.

a *R*_merge_ ​= ​∑_*hkl*_∑_*i*_|*I*_*i*_(*hkl*) [*I*(*hkl*)]|/∑_*hkl*_∑_*i*_*I*_*i*_(*hkl*), where *I*_*i*_(*hkl*) is the *i*th measurement of reflection *hkl* and [*I*(*hkl*)] is the weighted mean of all mea-surements.

b *R*_pim_ ​= ​∑_*hkl*_ [1/(*N* 1)] 1/2 ∑_*i*_|*I*_*i*_(*hkl*) [*I*(*hkl*)]|/∑_*hkl*_∑_*i*_*I*_*i*_(*hkl*), where *N* is the redundancy for the *hkl* reflection.

c *R*_work_/*R*_free_ ​= ​∑_*hkl*_| *F*_o_*F*_c_|/∑_*hkl*_|*F*_o_|, where *F*_c_ is the calculated and *F*_o_ is the observed structure-factor amplitude of reflection *hkl* for the working/free (5%) set, respectively.

### 2.2 Overall structure

**Fig. 1:**
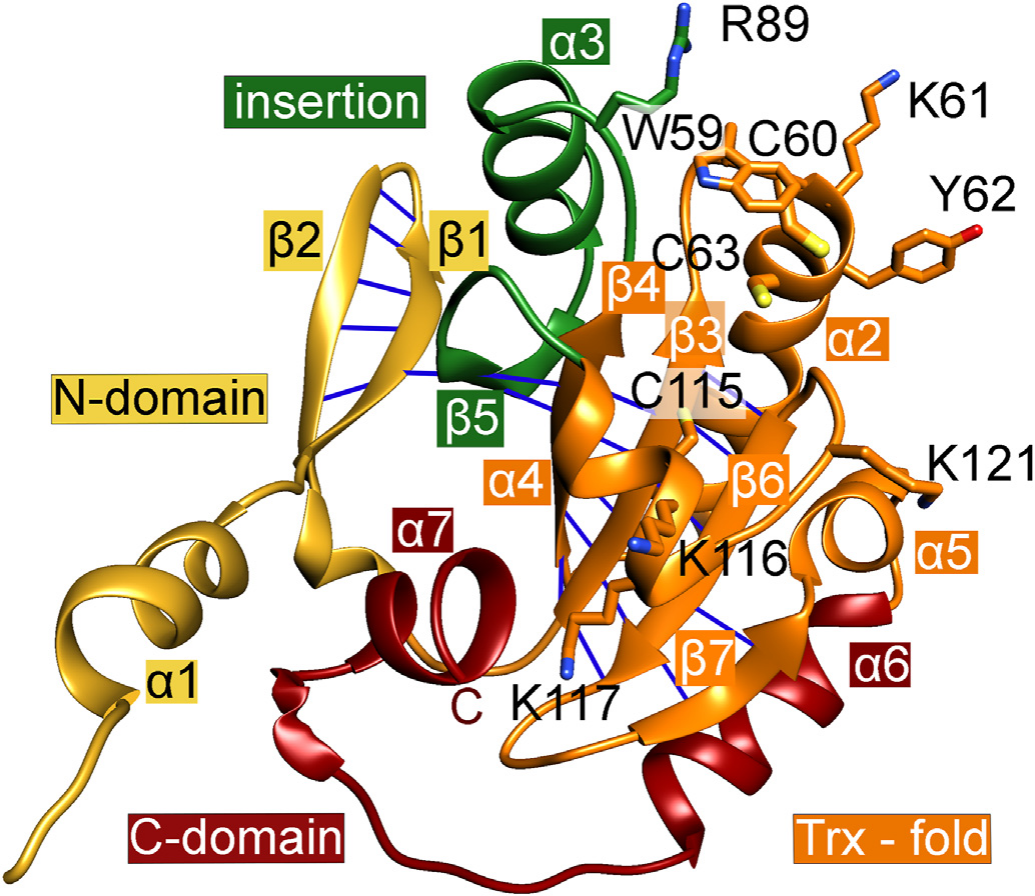
**Overall structure of the Plrx monomer.** The canonical thioredoxin fold is colored orange; the hydrogen bonds between the strands are indicated by blue lines.the active site residues (W59, C60, K61, Y62, C63) and the positively charged residues R89, K61, K116, K117 and K121 are shown as sticks, The insertion, which is not present in classic thioredoxin structures, is stained green, the N-terminal and the C-terminal domaine are colored gold and dark red. (For interpretation of the references to color in this figure legend, the reader is referred to the Web version of this article.)

The *Pf*Plrx monomer (12–179 aa) contains a twisted seven-stranded, mixed β-sheet (β1, β2, β5, β4, β3, β6, β7), surrounded by five α-helices (α2 to α5) (Fig. 1). The N-terminal region (1–50) comprises a helix–hairpin motif (α1, β1, β2) connected by a 3_10_ helix to the central domain. Furthermore, strands β6 and β7 form another hairpin at the C-terminal end of the sheet, followed by three helices (α5-α7). To facilitate comparison with the protein topology of other redoxins, we used the terminology ‘twisted’ seven-stranded β-sheet; although strands β5 (104–106) and β7 (135–137) are disturbed and connected to adjacent strands (β1, β4, β6) by only one or two H-bonds (Fig. 1). The longest helix (α2) in the *Pf*Plrx structure comprises five turns with the typical thioredoxin active site motive WCXXC (W59, C60, K61, Y62, C63) at its N-terminal end. Besides the redox-active cysteines, the *Pf*Plrx sequence contains two more *Plasmodium*-specific cysteine residues: The N-terminal cysteine residue C3 is not visible in the structure, and C115 is located at the exterior of helix α4 with a distance of 11 ​Å and 12.5 ​Å to C63 and C60, respectively.

**Fig. 2:**
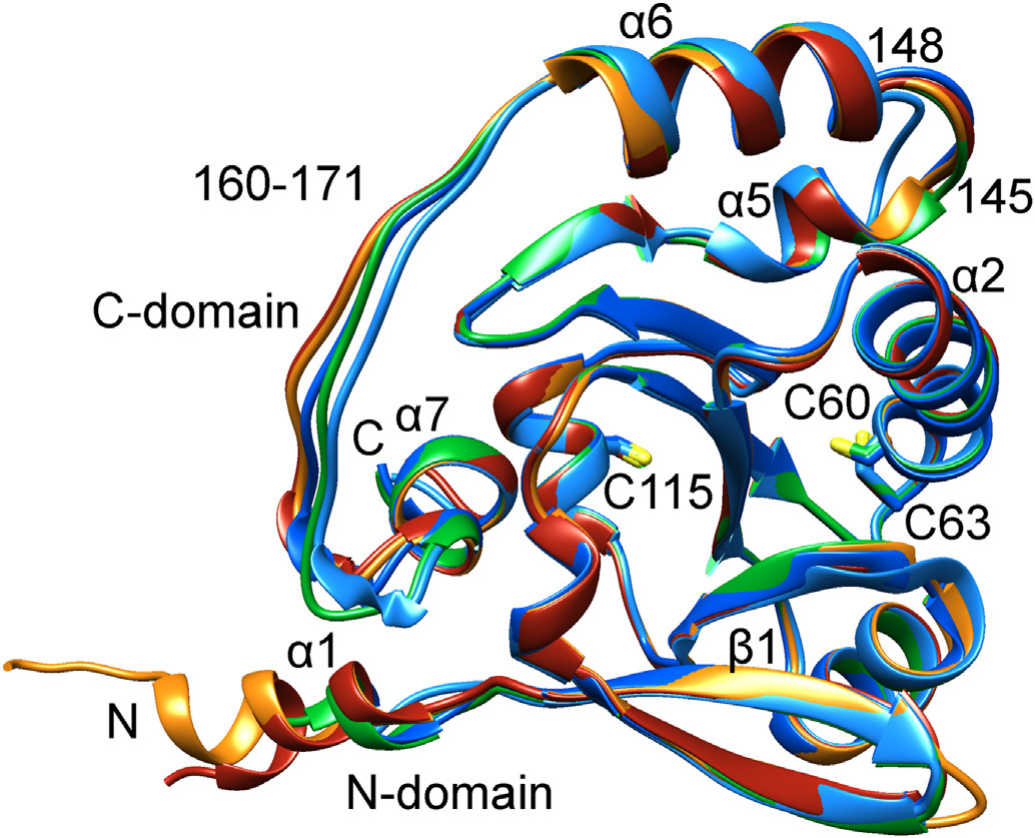
**Superpositions of (A) *Pf*Plrx monomer structures**. The structure that resulted from the hexagonal crystal is colored light blue; the four structures of the monoclinic crystal are colored dark blue, orange, green, and red. (For interpretation of the references to color in this figure legend, the reader is referred to the Web version of this article.)

The five independent *Pf*Plrx structures determined in the monoclinic (four monomers) and hexagonal (one monomer) crystal form are essentially similar (Fig. 2). Superimposition of the monoclinic structures reveals an rmsd between 0.5 and 0.7 ​Å with about 160 residues, and comparison with the hexagonal structure shows an rmsd of 0.9 ​Å with 153 residues. All structures are in the reduced form, with an equal distance (3.8 ​Å) between the Sγ-atoms of the active site cysteines (C60, C63). Minor differences occur at the N and C-termini and are probably due to crystal contacts at the loop regions 145–148 and 160–171. The number of N-terminal residues seen in the electron density varies among the five structures (Fig. 2). Furthermore, the N-terminal helix α1 is missing in the hexagonal structure.

### 2.3 Comparison with other redoxins

**Fig. 3:**
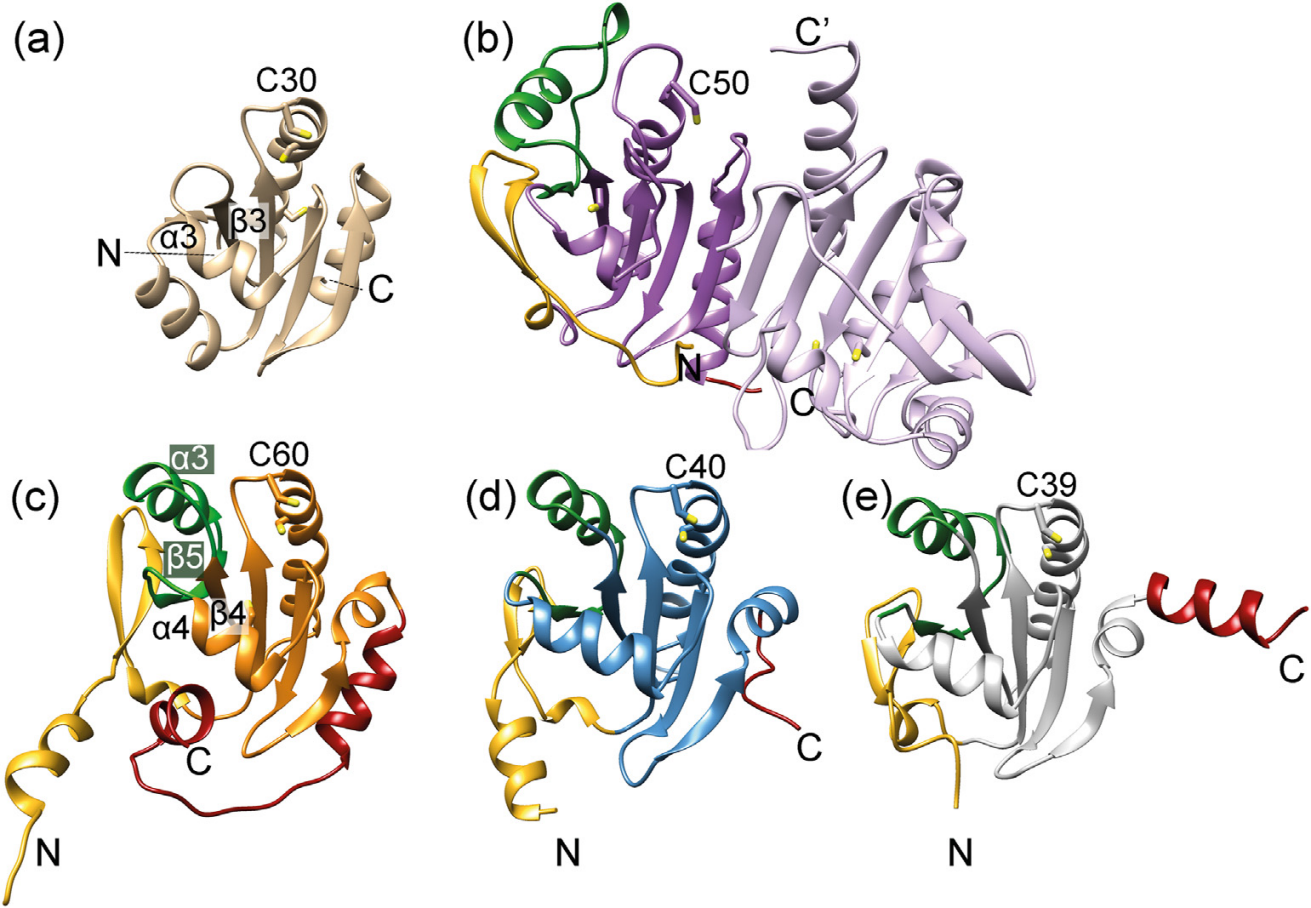
**Structure variations in the thioredoxin superfamily.** The C-terminal domain is colored dark red, and if present, the N-terminal domain and insertion are highlighted in gold and green, respectively. The cysteine in the active site, which forms a mixed disulfide with the target enzyme, is labeled. (a) Monomer structure of *Pf*Trx (**2mmo**). (b) Dimer structure of *Pf*Prx1m (**2c0d**), one subunit is stained purple, the other light purple. (c) Monomer structure of *Pf*Plrx. (d) Monomer structure of *Lm*Txn (**3s9f**) (e) Monomer structure of *wb*Trx (**4fyu**). (For interpretation of the references to color in this figure legend, the reader is referred to the Web version of this article.)

Sequence alignment (Sievers et al., 2011) of *Pf*Plrx with other thioredoxins (Trx), peroxiredoxins (Prx), or tryparedoxins (Txn) revealed sequence identities of 23% or less*.* But the structural comparison with other redoxins confirms that *Pf*Plrx belongs to the thioredoxin superfamily (Becker et al., 2003) and shows that *Pf*Plrx adopts a modified thioredoxin fold. The classic thioredoxin fold (Holmgren et al., 1975; Katti et al., 1990; Menchise et al., 2001) comprises a four-stranded β-sheet surrounded by three α-helices (α2, α4, α5) forming one compact domain with a βαβαββα topology (Fig. 3a).

It is known that the sequences in the giant thioredoxin superfamily are highly divergent (Atkinson and Babbitt, 2009; Carvalho et al., 2006). Therefore, we searched for similar structures in the Protein Data Bank using the Dali server (Holm, 2020). The highest match was found with tryparedoxin structures. (Z-score 15.4 to 16.6, rmsd 1.7 to 2.2 ​Å, seq_id 17–23% with 143–152 residues). All other structures, including the classical Prx structures and Trx structures reach highest Z-scores of 11, except for an atypical Trx structure from *Wuchereria bancrofti* (**4fyu**, Nasser, Y. et al. unpublished work) with a Z-score of 15.0 (rmsd 2.0 ​Å, seq_id 21%). Despite the similarity of the secondary structure elements between the atypical Trx and Plrx, the best structural superimposition for the whole enzyme is found with Txn (Fig. 3).

In Trx and similar in Grx, strand β3^Trx^ and helix α3 ^Trx,^ are linked via a short turn, comprising four residues (Fig. 3). In Plrx the corresponding structural elements are connected by a 26-residue long insertion, containing an α-helix (α3) and a β-strand (β5). This insertion is also present in tryparedoxin ((Wagner et al., 2019; Alphey et al., 1999; Fiorillo et al., 2012), furthermore Plrx and Txn share the N-terminal domain, which is also not present in Trx or Grx. These two structural elements also occur in 1- and 2-cys peroxiredoxins, but are arranged completely differently compared to Txn and Plrx. Consequently, only about ninety residues can be structurally superimposed (rmsd 3 A) with the Prx1m structure (Boucher et al., 2006), moreover, the amino acid sequence matches only to a maximum of 16%, both together result in poor Z-scores below 8.6. However, neither the sequence identity of 23% nor the Z-score of 16 are particularly high, Plrx and Txn share the most similarities within the Trx superfamily. Exclusive to Plrx is the large C-terminal domain containing two helices, which is not found in this form in other members of the Trx superfamily. (Fig. 3)*.*

### 2.4 Active site

The active site 59–63(WCKYC) is located at the N-terminal end of helix α2 (Fig. 1, Fig. 2). The N-terminal cysteine residue (C60) is exposed on the surface of the protein, located on a loop, which connects β3 with the active site helix α2, while the second cysteine is part of this helix. In other redoxins, the solvent-exposed cysteine residue forms a mixed disulfide with target proteins (Kallis and Holmgren, 1980). The second cysteine (C63^Plrx^) subsequently attacks this mixed disulfide, thereby releasing the reduced target protein and the oxidized enzyme (*Pf*Plrx). The environment of the active site, comprising *Pf*Plrx residues F55–N71 (sections of β3 and α2) and C115–F126 (sections of α4 and β6), is structurally conserved in the thioredoxin superfamily (colored red in Fig. 1). For this small region (26 aa) the sequence homology is significant for tryparedoxin (Txn) but still in the low thirties for *Wb*Trx (Nasser, Y. et al. unpublished work), *h*Trx (Smeets et al., 2005), *Pf*Trx (Fritz-Wolf et al., 2013), and *Pf*Grx (Yogavel et al., 2014). Notably the active site sequence differs: WCKYC in Plrx, WCPPC in Txn, WCPYC in Grx, and WCGPC in Trx. Except for Plrx, the active site of the proteins listed above contains a proline, which is often found at the curvature of an amino acid chain. However, despite the lack of proline, the backbone dihedral angles of the Plrx CXXC motif are consistent with those typically observed for active sites in the thioredoxin superfamily (Fig. 3).

### 2.5 Similarities and differences between Plrx and Txn

It is known that the 3-D structure is much better conserved than the sequence (Illergard et al., 2009). Members of a superfamily have large divergent sequence similarity but the same primary function. Within the Trx superfamily, Plrx has the best match with Txn, both structurally and in terms of sequence identity in the active site region. Therefore, we hypothesize that they are more closely related within the thioredoxin superfamily than other members and may define an extra subgroup consisting of Plrx and Txn. In both oxidoreductases, the β-sheet is twisted, and the N-terminal β-hairpin is connected only via one (Plrx) or two hydrogen bonds (Txn) to the central 5-stranded β-sheet (Fig. 1, Fig. 3). In addition to the few hydrogen bonds between the N-terminal β-strands and the central β-sheet, the N-terminal region is also connected to the central domain by a 3_10_ helix followed by a loop. The main structural features that distinguish Plrx and Txn from other members in the superfamily are the N-terminal hairpin and the insertion between β4^Plrx^ and α4^Plrx^ (Fig. 3).

**Fig. 4:**
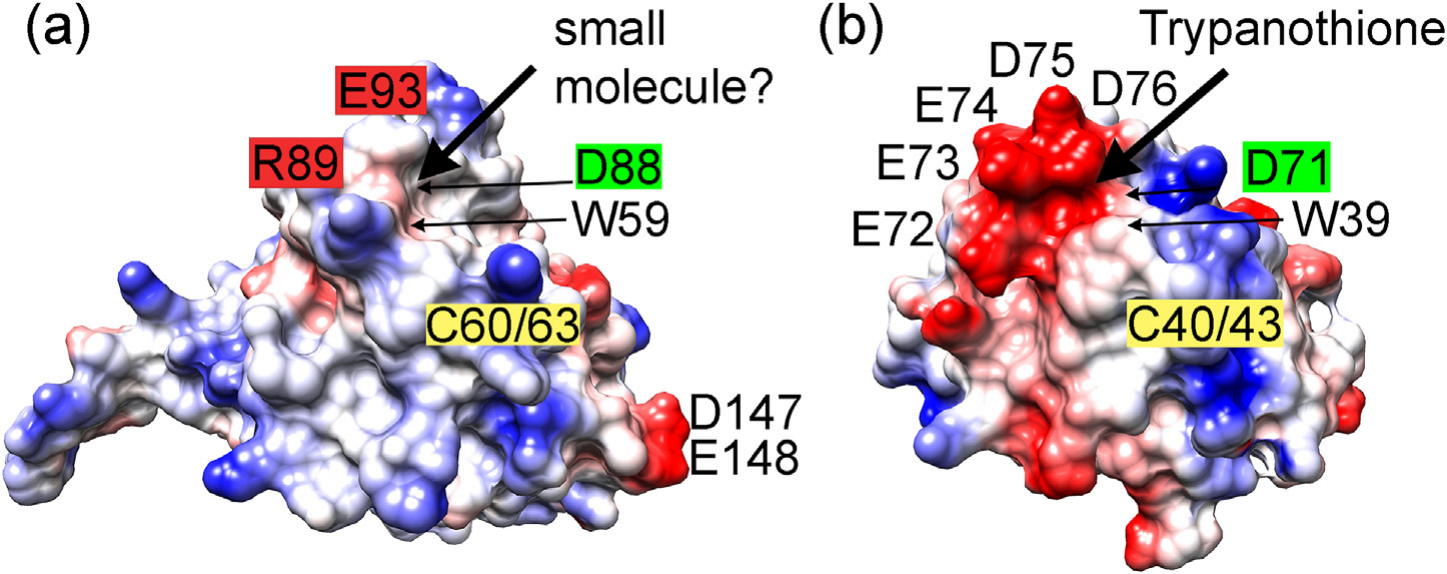
**Putative binding sites in *Pf*Plrx or *Lm*Txn.** (a) Surface representation of *Pf*Plrx; residues E93 and R89 interact via a salt bridge. (b) Surface representation of *Lm*Txn (**3s9f**)**.** The molecular surfaces are color-coded according to electrostatic potential (red −10 to blue 10). (Chimera package). For orientation, some residues including the active site WCxxC are labeled. (For interpretation of the references to color in this figure legend, the reader is referred to the Web version of this article.)

It is supposed that this insertion serves to bind Try, which reduces Txn (Alphey et al., 1999). The proposed binding site is close to the active site of Txn, comprising a cluster of negatively charged residues (70–76, WDEEEDD), suitable for binding positively charged Try (Fig. 4) (Alphey et al., 1999). As mentioned above, the structural similarity with the homologous region in Plrx is very high, but the surface charge is not (Fig. 4). In Plrx, there are only two negatively charged residues in this region, D88 and E93. In addition, the access to the potential binding pocket is blocked by a hydrogen bond between E93 and R89. The structural similarity between the putative Try binding pocket with the homologous region in Plrx indicates that this structural element specific to *Plasmodium* and kinetoplastids could be used to bind small redox partner molecules in both proteins. The access blockage of the binding pocket by the interaction between E93 and R89 could be easily resolved, allowing access. Low molecular weight substrates (TCEP, DTT), including trypanothione but not glutathione, are able to reduce *Pf*Plrx (Becker et al., 2003). However, Try is not present in *Plasmodium*, TCEP or similar molecules are not present in the cell, thus until now we don't know suitable small redox molecule.

Another difference to Txn is the distinct C-terminal domain. Along these lines, in contrast to Txn and Trx, the Plrx structure contains only small, negatively charged surface regions: one at the N-terminal helix (E19, E21) and one at the loop connecting the C-terminal helices α5 and α6 (D147, E148). Plrx and Txn adopt a very similar fold, but the charge distribution on the surface differs strongly. As shown in Fig. 4, the surface of Plrx is mainly neutral or positively charged, indicating either different redox partners than those of Trx or Txn, or the partners have adjusted their surface charges accordingly.

### 2.6 Comparison with the human Trx dimer and the TrxR-Trx complex

**Fig. 5:**
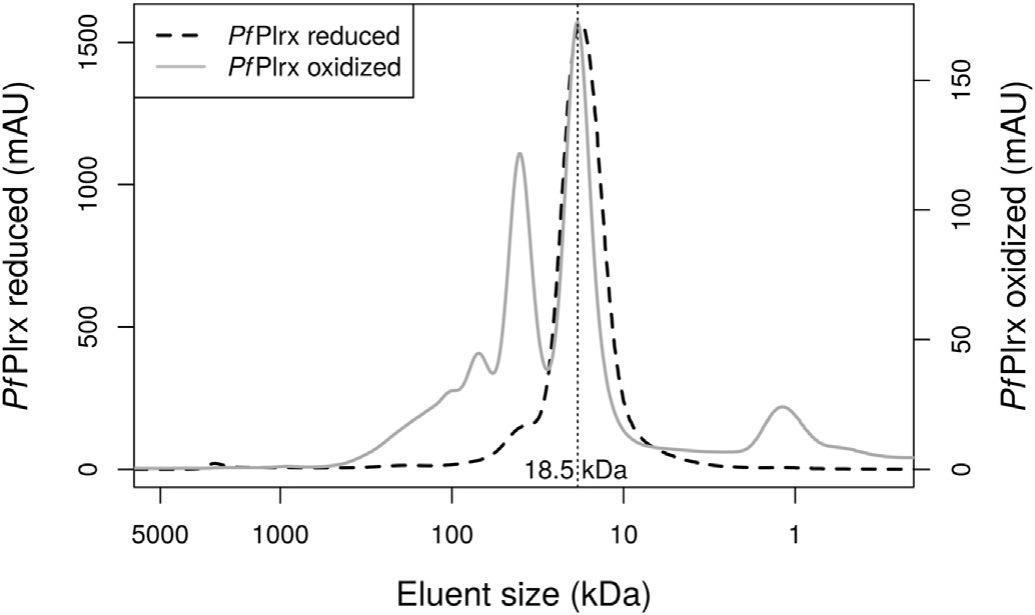
**Elution profile of *Pf*Plrx depending on its oxidization state**. In size-exclusion chromatography (SEC), *Pf*Plrx exhibited considerably different elution profiles depending on its oxidization state. *Pf*Plrx, which was reduced by adding Tris(2-carboxyethyl)phosphine (TCEP) (black dashed line), displayed a single peak at 18.5 ​kDa, which is in close agreement with the calculated weight of the monomeric protein of 22 ​kDa. *Pf*Plrx that was at least partially oxidized due to a lack of TCEP eluted in two main peaks. One peak corresponded to the weight of the monomer, and the second was equivalent to the dimeric protein. The scales vary due to different amounts of protein used in both experiments.

We determined the oligomerization state of the *Pf*Plrx wild type enzyme with size-exclusion chromatography. In its oxidized form, the enzyme eluted in two main maxima (Fig. 5), with one peak corresponding to the weight of the monomer and the other to that of the dimer. Adding a reducing agent to the protein solution leads to the removal of the dimer peak, most likely by dissolving a disulfide bridge. Therefore, two cysteine residues, each from one subunit of the dimer, must be involved in dimer formation.

**Fig. 6:**
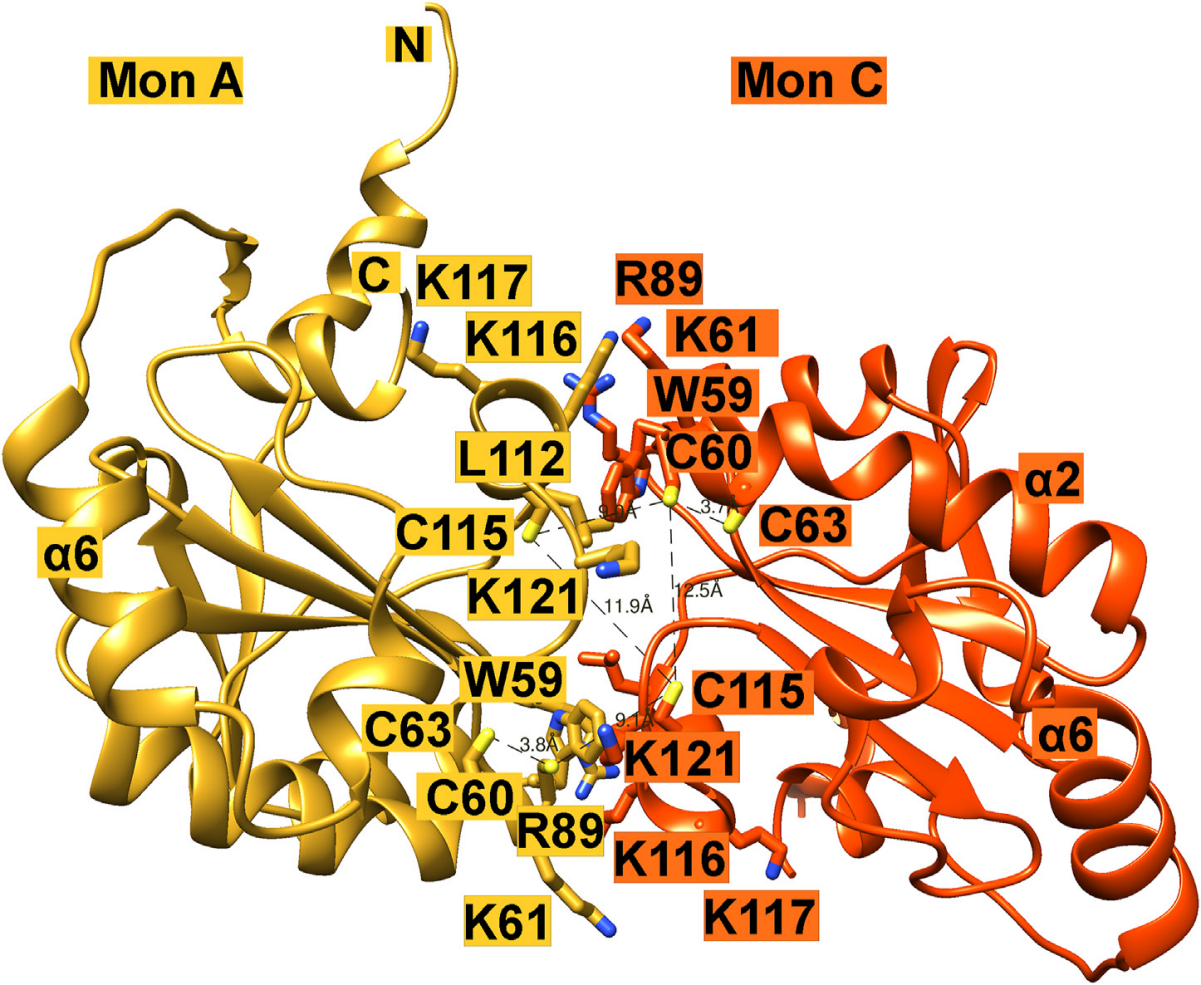
**Monomer contacts in the asymmetric unit of the monoclinic crystals.** Dimer AC (yellow, orange) and its relevant interface residues are drawn with sticks and colored according to the subunit color. (For interpretation of the references to color in this figure legend, the reader is referred to the Web version of this article.)

**Fig. 7:**
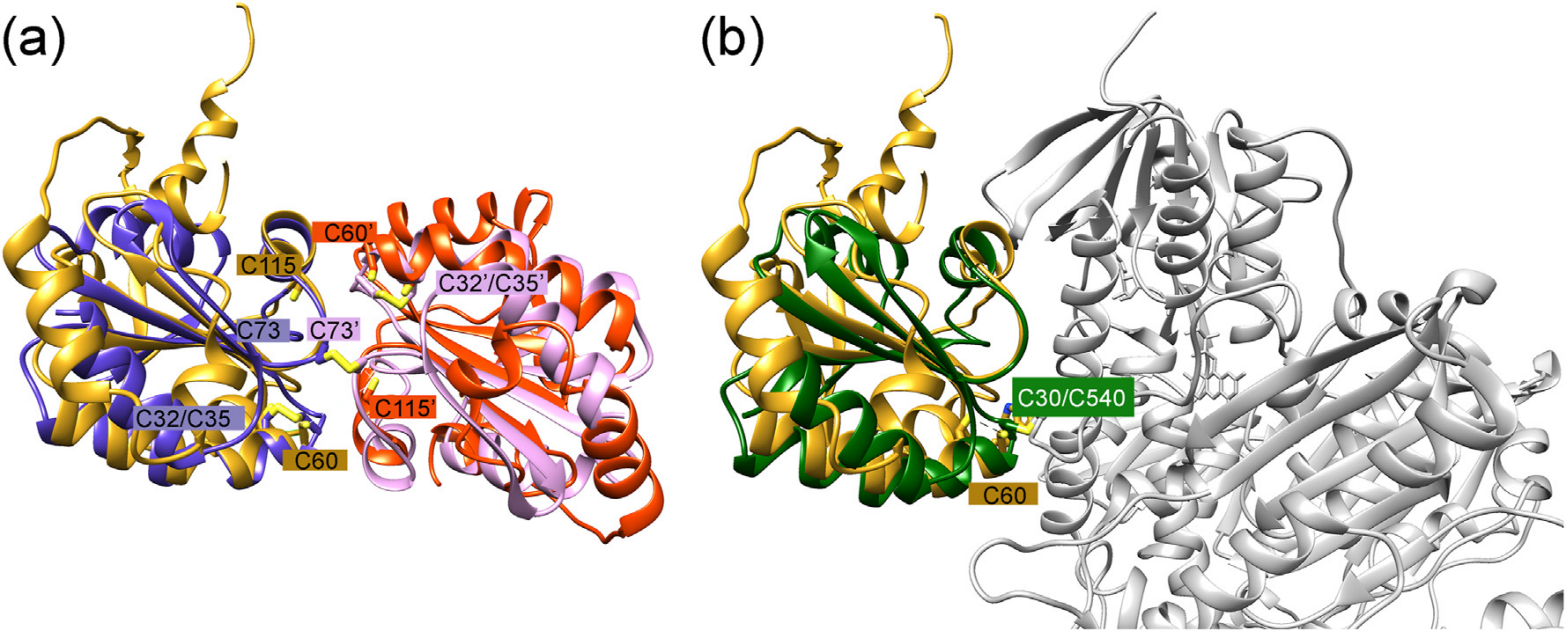
**Interfaces (A) Superimposition of the *Pf*Plrx-with the *h*Trx—dimer**. The Plrx dimer is shown in the same orientation as in Fig. 6. The two subunits of *Pf*Plrx or *h*Trx (**1eru**) are colored gold and red, or purple and pink, respectively. Active site cysteine residues and cysteine residues involved in the dimer interface are labeled. Residues of the second subunit are marked with an apostrophe. **(B) Superimpostion of the *Pf*Plrx-dimer with the *Pf*Trx-*Pf*TrxR -complex (4J57**). For clarity only one subunit (gold) of the Plrx dimer is shown, *Pf*Trx is colored green and *Pf*TrxR grey, active sites are marked by C60 (Plrx) and C30/C540 (*Pf*Trx-*Pf*Trxr). (For interpretation of the references to color in this figure legend, the reader is referred to the Web version of this article.)

We obtained three different types of dimers in our crystals. The asymmetric unit of the hexagonal crystal form contains one monomer, thus the dimer AA′ is formed by crystal contacts. The four monomers (A, C, B, D) in the asymmetric unit of monoclinic crystals form two types of dimers: Dimer AC (Fig. 6) and Dimer AD (not shown). It is worth mentioning that only one (AC) of the three dimers contains cysteine residues (C60, C60′, C115, C115′) at the interface. The active site residues (W59, C60, K61), helix α4 residues (112–117), loop residues 120–123 and R89 form the dimer interface. There are four lysine residues (K61, K116, K117, K121) and one arginine residue (R89) at the interface, but they do not form polar bonds between the subunits. Instead, they use the aliphatic part of their side chains for nonpolar interactions (Fig. 6). Overall, the interactions between the subunits are mainly hydrophobic. As mentioned earlier, we have crystallized *PfP*lrx in its reduced form so that none of the cysteine residues at the interface, C60 and C115, and C60′ and C115′ of the adjacent subunit form a disulfide. The distances between C115 Sγ - C115′ Sγ, and C115 Sγ - C60’ Sγ are 12 ​Å and 9 ​Å, respectively. However, a change in the side chain conformation of C60 and C115′, which reduces the distance between the Sγ atoms to 4.6 ​Å, and a slight change in conformation of the amino acid chain would facilitate a disulfide between C60 and C115’ (Fig. 6, Fig. 7a).

Dimer formation via a disulfide bridge is also known from *human* thioredoxin 1 (Weichsel et al., 1996). This intermolecular disulfide is formed by C73 and C73′, and mutation of one of these cysteines is sufficient to prevent dimer formation. This is similar to the Plrx dimer, where the addition of a reducing agent to the protein solution leads to the removal of the dimer peak in size exclusion chromatography. Dimer formation without implication of any cysteine residue are seen in *h*Trx2, instead there are mainly hydrophobic contacts (Smeets et al., 2005).

In the case of both *h*Trx and Plrx, dimer formation leads to an inactive redoxin because the redox-active cysteine is hidden in the dimer interface, so interaction with redox partners would be impossible. The physiological role of the inactive human thioredoxin dimer is still unknown (Kallis and Holmgren, 1980). It is believed that dimer formation may function as part of a regulatory capacity (Weichsel et al., 1996; Ren et al., 1993). In a recently published study (Campos-Acevedo et al., 2017), the authors suggested that dimerization is a common feature of thioredoxins from humans and other eukaryotes, but the interfacial residues are poorly conserved among members of the thioredoxin family (Eklund et al., 1991). Campos-Acevedo et al. (2017) point out that the residues involved in dimerization are also involved in the interaction with target enzymes (Saitoh et al., 1998; Nishiyama et al., 1999; Ueno et al., 1999). In line with these findings, the structural superimposition of the Trx dimer with the Plrx dimer AC shows that corresponding regions of both enzymes are involved in dimer formation (Fig. 7 A).

The superposition of the *Pf*Plrx dimer with the 3D structures of *h*TrxR (Fritz-Wolf et al., 2011) or *Pf*TrxR (Fritz-Wolf et al., 2013) in complex with *h*Trx and *Pf*Trx, respectively, show that exactly these interface residues are involved in complex formation (Fig. 7 B). The structural comparisons of Plrx with the Trx dimers and with the TrxR-Trx complex structures strongly suggest that the interface residues of the Plrx dimer are used for complex formation with the target proteins.

**Fig. 8:**
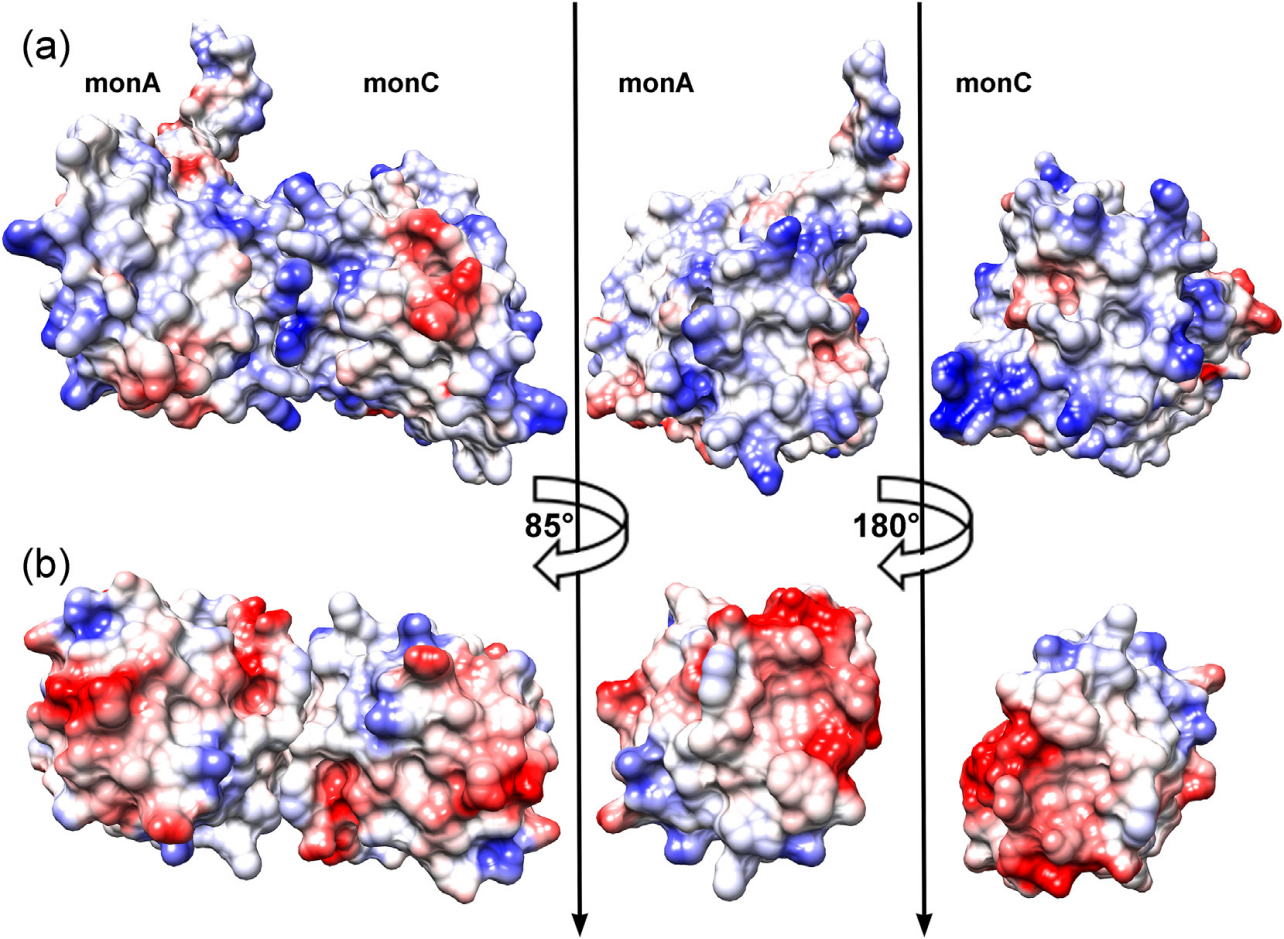
**Comparison of the charge distribution in the dimer interface of *Pf*Plrx and *h*Trx**. To show the interaction surface of the two subunits, we rotated the dimer by 85°, putting the surface of subunit C in front of the surface of subunit A. We therefore show only subunit A. This dimer position is then turned by 180°, consequently subunit C is now on the bottom, and we display subunit C. Panel A shows the surfaces of the *Pf*Plrx dimer AC. Panel B shows the surfaces of the *h*Trx dimer.

The distribution of charge on the contact surface is very different for the three proteins: *h*Trx and *Pf*Trx are negatively charged and Plrx is positively charged. (Fig. 8). The positively charged surface of this region in Plrx with five positively charged residues (K61, R81, K117, K117, K121) indicates different target enzymes than those of Trx. For example, it could bind via the lysines to the cell membrane or to plasminogen, which is known to bind to bacterial proteins via lysine residues. Plasminogen deficiency leads to reduced reactivity towards excessive clotting activity.

### 2.7 Functional considerations

It is known that dithiols such as Trx, Grx, and Txn and a few low-molecular substances such as Try, DDT, and TCEP can reduce Plrx, and the best reducing agents so far are Txn from *Trypanosoma brucei* and Grx from *Plasmodium falciparum* (Becker et al., 2003). Until now, no enzymatically catalyzed reduction of Plrx has been identified except for rather unspecific disulfide dithiol exchange reactions. However, Plrx is active in an insulin reduction assay and serves as a physiological electron donor for Prxs (Nickel et al., 2005) and ribonucleotide reductase.

Under intracellular reducing conditions, the majority of Plrx molecules are likely present as monomers. Under oxidative challenge, Plrx dimers could form, which might have a redox-regulatory function: The active site of the inactive Plrx dimer is not accessible to reduction anymore. This would favor other redox relays in the cell, such as Trx and Grx dependent reactions, which might be more essential for survival under stress conditions. Until now, no Plrx-specific reductase has been discovered; therefore, dimer formation is perhaps even a precondition for Plrx to react with its reducing agent. Initially, the dimer is dissolved, and then the monomer is reduced. Oxidative stress could thus cause dimer formation as a regulatory mechanism for the biological activity of Plrx. Overall, the entire Plrx surface is much more positively charged than the surface of Trx or Txn (Fig. 4, Fig. 8), and so Plrx might have additional binding partners than those known so far.

## 3 Conclusions

Structural analyses confirmed that PfPlrx belongs to the thioredoxin superfamily, despite the highly divergent sequence to other members and the modest results of a search for similar structures using the Dali server. Unique to Plrx, is the distinct C-terminal domain, containing two helices. The highest relationship in terms from sequence and structural fold is found with Txn.

We therefore propose a new subgroup within the thioredoxin superfamily with the following common characteristics: 1.) They are larger than classical Trx. 2.) They form a ‘pseudo’ seven-stranded β-sheet, with an N-terminal domain, containing a helix-hairpin motif (α1, β1, β2) connected by a 3_10_ helix to the central domain; the orientation of this small domain (ca. 50 aa in Plrx) is flexible to the central domain. 3.) There is a large insertion within the central domain between the β-strand (β4^Plrx^) and one of the surrounding helices (α4^Plrx^). For Txn, this insertion includes a binding pocket for Try; no corresponding molecule is yet known for Plrx. 4.) So far, an atypical Trx from *Wuchereria bancrofti* (**4fyu**) (and all known tryparedoxins (*Lm, Tb,* Cf) are members of this new subgroup. The members fulfill the typical thioredoxin family functions, and just like Txn, Plrx is able to provide electrons to ribonucleotide reductase (Krauth-Siegel et al., 2003).

The surface of Plrx is mainly neutral or positively charged, in contrast to Trx or Txn with more negatively charged areas. Therefore, we expect either different redox partners for Plrx than for Trx or Txn, or the partners have adjusted their surface charges accordingly. Moreover, we were able to show that Plrx is able to form dimers, depending on the redox level of the environment. We suppose that the dimers are inactive because the interface residues, comprising the redox active cysteine, are presumably necessary for complex formation with redox partners. We do not know whether the dimer has a regulative function, but we suppose that the residues located at the dimer interface interact with target proteins. Moreover, these interface residues correspond to the interface residues of the *h* and *Pf*TrxR-Trx complexes. In particular, we demonstrated that the dimeric interface residues of Plrx are most likely involved in complex formation with redox partners. Notably, five positively charged amino acids contribute to the dimer interface in Plrx and are suitable for interacting with the cell membrane or plasminogen. This would demonstrate new putative redox functions in a member of the thioredoxin superfamily. However, it would be very interesting to combine site-directed mutagenesis of the active-site cysteines with further biochemical analyses to study dimer formation in more detail.

Since the surface charge of plasmoredoxin differs largely from other members of the thioredoxin superfamily (*Lm*Txn, *h*Trx, *Pf*Trx), this initial 3-D structure might serve for designing specific redox modulatory compounds for *Plasmodium*.

## 4 Methods

### 4.1 Protein preparation

Plrx cloning has already been described (Becker et al., 2003). For protein production, Qiagen's expression system consisting of a pQE30 vector and M15 *E. coli* cells was used. After inoculation with a 50 ​ml starter culture, cells were grown in 1 ​L of LB medium containing 50 ​μg/ml kanamycin for 4 ​h at room temperature. Ni-NTA-based affinity chromatography was used to purify. The purification buffer consisted of 300 ​mM NaCl and 50 ​mM Tris pH 8.0. To prevent protein dimers from forming, 1 ​mM TCEP had to be added to the eluate. Size exclusion chromatography (SEC) further increased purity and assured conformational homogeneity of Plrx (column: HiLoad 16/60 Superdex 200, flow rate: 1 ​ml/min). Protein fractions with approximately 99% purity, as judged via SDS-PAGE, were again supplemented with 1 ​mM TCEP, pooled, and concentrated using Vivaspin 20 centrifugal concentrators with a 10,000 ​Da MWCO.

### 4.2 Crystallization, data collection, and processing

We obtained monoclinic (space group P2_1_) and hexagonal crystals (space group P6_1_) of Plrx from *P. falciparum.* The hexagonal crystals were grown at 25 ​C with the hanging drop technique. The protein was concentrated to 15 ​mg/ml and stored in a buffer of 0.05 ​M sodium phosphate (pH 8.0), 300 ​mM NaCl, and 2 ​mM DDT. We mixed 2 ​μl of protein buffer solution, 2 ​μl of 1.6 ​M ammonium sulfate (AS) and 1 ​μl of 1 ​M glycine in the drop. The first crystals were found one year later and were soaked in a cryo buffer with 30% glycerol, 2.8 ​M AS, and 0.5 ​M glycine before the measurements. The monoclinic crystals were produced using a Honeybee 961 crystallization robot. Plrx was concentrated to 30 and 40 ​mg/ml (buffer: 50 ​mM Tris (pH 8.0, HCl), 300 ​mM NaCl, 1 ​mM TCEP), and 200 ​nl of the protein solution was mixed with 200 ​nl of precipitant solution in a sitting drop setup. Useable crystals grew from screen JCSG II (Qiagen), well D6 (0.1 ​M sodium citrate pH 5.5, 40% PEG 600) within 5 days. Further crystals were grown via grid screening around the initial conditions (32–46% PEG 600, sodium citrate pH 4.5–6.0, with 0.05, 0.1, and 0.15 ​M). Several of those conditions yielded diffraction quality crystals with similar resolutions.

Diffraction data of the monoclinic crystals were collected at the X10SA beam line of the Swiss Light Source in Villigen, Switzerland; the hexagonal crystals were collected in 2003 ​at beam line ID14-4 of ESRF. Diffraction data were collected at 100 ​K and processed with XDS (Kabsch, 2010). Both crystal types, monoclinic (space group P2_1_) and hexagonal (space group P6_1_), diffracted to 1.6 ​Å (Table 1). The asymmetric unit of the monoclinic and hexagonal crystal types contained four and one monomers of Plrx, respectively. During refinement, 5% of all reflections were omitted and used for calculating an Rfree value.

### 4.3 Structure determination

Attempts to solving the hexagonal structure via the automatic molecular replacement method (Adams et al., 2010), using all hits found with NCBI Blast as start models, were unsuccessful. An initial partial solution was revealed with an automatically modified *Wb*Trx model (4fyu). This solution contained several fragments; overall, only 100 amino acids of 179 Plrx residues could be placed. Refinement of this poor model resulted in an R_free_ of 48% with an uninterpretable electron density.

We soaked the monoclinic crystals with thiomersal. The resulting dataset and the native dataset were merged with XSCALE (Kabsch, 2010). The mean anomalous difference in units of its estimated standard deviation (|F(+)-F(−)|/Sigma) was 2.5 or 1.5 and the anomalous correlation (=percentage of correlation between random half-sets of anomalous intensity differences) was 84 or 51 ​at a resolution of 6.2 ​Å and 3.1 ​Å, respectively. Using AutoSol (Adams et al., 2010) with the monoclinic native data, the anomalous scattering information of the thiomersal dataset (sad peak, sites) and the partial solution of the hexagonal dataset as input files, produced another partial solution (ca. 100 aa) with 11 fragments and 152 waters. The figure of merit (FOM) value of this solution was 0.54. Although some amino acid fragments were wrongly assigned to the electron density, the reasonable electron density could be used for manual rebuilding and subsequent refinement. In the first refinement with the corrected structural model and the high-resolution data set, the R_free_ value dropped from 44.7% to 24.4%. After several cycles, we revealed a complete model of Plrx. Data collection and refinement statistics are shown in Table 1.

The PHENIX program suite (Adams et al., 2010) served for reflection phasing and structure refinement. The interactive graphics program Coot (Emsley et al., 2010) was used for model building. The sequences were aligned using Clustal Omega (Sievers et al., 2011). The structures were superimposed using the SSM algorithm tool (Krissinel and Henrick, 2004), implemented in the Coot graphics package. The SSM tool is a structural alignment based on secondary structure matching. Molecular graphics images were produced using the UCSF Chimera package (Pettersen et al., 2004).

## Accession numbers

Coordinates and measured reflection amplitudes have been deposited in the Brookhaven PDB. PDB ID: 7AOJ (hexagonal form) and PDB ID:7AOO (monoclinic form).

## Conflict of interest

The authors declare no conflict of interest.

## Author contributions

KFW solved the X-ray structure, analyzed data, and wrote the manuscript, JB crystallized the enzyme and carried out the oligomerization studies. SR and KB monitored the biochemical experiments, KB contributed to writing the manuscript and was the principal investigator. All authors have critically discussed the results and carefully read the manuscript.

## Declaration of competing interest

The authors declare no competing interests.
